# Testicular and Related Size Evaluations in Nigerian Sahel Goats with Optimal Cauda Epididymal Sperm Reserve

**DOI:** 10.1155/2015/357519

**Published:** 2015-12-08

**Authors:** Y. Abba, I. O. Igbokwe

**Affiliations:** ^1^Department of Veterinary Pathology, Faculty of Veterinary Medicine, University of Maiduguri, PMB 1069, Maiduguri 600233, Borno State, Nigeria; ^2^Department of Veterinary Pathology and Microbiology, Faculty of Veterinary Medicine, Universiti Putra Malaysia, 43400 Serdang, Selangor, Malaysia

## Abstract

Testicular sizes of animals are important for identification of those with adequate sperm production. The aim of this study was to define the testicular and related size estimates that would be associated with optimal cauda epididymal sperm counts (ESC) in Sahel goats based on postmortem evaluations. A stratified quota sample population of 125 male goats inclusive of all testicular sizes was taken at a slaughterhouse in Maiduguri, Nigeria. The bucks were aged 18–30 months and weighed 17.04 ± 2.99 (12–25) kg. Body, testicular, and epididymal weights of each goat with other related size measurements were estimated. ESC was determined from homogenized tissue using a manual cytometer. At the cut-off ESC of >1.1 × 10^9^ sperm heads, 66 (52.80%) of the goats had optimal ESC which was associated with testicular weight of 59.90 ± 16.10 (31.40–86.20) g, gonadosomatic index of 3.51 ± 0.69 (2.00–4.50) g/kg, and scrotal circumference of 19.07 ± 1.29 (17.00–21.80) cm. The size variables of the scrotum and testis correlated with one another and with the ESC. These findings provide data that may be used to anticipate adequate antemortem sperm reserve based on testicular size during preliminary selection of sires for breeding from a sexually mature Sahel buck population.

## 1. Introduction

The breeds of goats in Nigeria are either dwarf (West African Dwarf type, WAD) or long-legged (Sahel type). The ecotypes of Sahel goats in northern Nigeria are Borno White (BW), Kano or Savannah Brown (KB), and Red Sokoto (RS), with no morphological features for external differentiation apart from coat color [1–5]. Investigations of Sahel bucks to establish baseline data required for evaluation of their reproductive health are scanty, but reports are available on their potential for sperm production [6–16].

Normospermia could be anticipated when cauda epididymal sperm reserve is adequate, since the sperm output in the semen depends on the quantity of sperm stored in the cauda epididymides [17]. Reference values of testicular size and sperm cell counts in cauda epididymides of Nigerian goats at postmortem or after castration, based on adequate number of reference individuals, were not accessible, but such values ought to be available to indicate normal variations observed in healthy populations so that it would be possible to identify individuals having abnormal values in unhealthy conditions [18]. Four postpubertal WAD goats at 5 months of age weighing 8.90–11.00 kg in the early dry season had cauda epididymal sperm reserve of 1.57 ± 0.32 (1.20–1.90) × 10^9^ [9]. At the age of 12–14 months and weighing 19.52 ± 1.25 kg, 15 WAD goats were reported to have cauda epididymal sperm reserve of 1.46 ± 0.26 (1.20–1.70) × 10^9^ [15]. The WAD goats, in these reports, were intensively managed and fed adequately with supplements to obviate the effect of undernutrition on reproductive capacity [9, 15] and could be presumed to represent reproductively sound animals in the population. There is no evidence that the potential for sperm production differed between WAD and Sahel breeds [19]. Increasing age and body weight positively correlated with testicular sizes of goats [20–23]. Larger testes correlated with their scrotal circumferences [19, 24–26]. Testicular and cauda epididymal sperm reserves positively correlated with testicular weights and scrotal circumferences in RS goats [8]. 

The Sahel goat has adequate sperm production at 3 months of age with sperm cell concentration increasing with age [11]. The quality of sperm was reported to improve with age and later diminish with ageing of the buck beyond 30 months, but sperm quality was better in the dry than wet season [12, 16]. Bilateral testicular hypoplasia [27, 28] and atrophy [28, 29] associated with small-sized testes and aspermatogenesis occur among Sahel bucks [30]. Unilateral cryptorchid Sahel bucks had similar sperm concentration with normal bucks, but the percentage of sperm cell abnormalities was higher than normal [31]. Breeding programmes require assurance of the reproductive capacity of sires, but in areas with low technical and laboratory support for semen evaluation, farmers may need a reference range of testicular sizes, without extraneous mitigating conditions, which may be associated with adequate sperm reserve for reproductive efficiency.

In this study, the testicular sizes of sexually mature Nigerian Sahel goats were evaluated with their scrotal and epididymal sizes and testicular and cauda epididymal sperm counts with the aim of identifying goats with optimal sperm output from testicular and related size variables.

## 2. Materials and Methods

One hundred and twenty-five apparently healthy male Sahel goats in good body condition aged 18–30 months and weighing 10–25 kg, presented for slaughter at the metropolitan abattoir, Maiduguri, Nigeria, were selected by stratified quota sampling to capture those with various testicular sizes from small to large sizes with bilateral symmetry and the right-sided testis was consistently used for the study. They were aged by dental examination and weighed. The scrotal length (SL) and circumference (SC) were estimated with measuring tape. The testes (with their epididymides), with bilateral symmetry and without apparent lesions other than varying size, were collected and transported in an ice pack to the laboratory where the testes and epididymides were separated by dissection and weighed. The gonadosomatic index (GSI, g/kg) was estimated as the ratio of each testicular weight to body weight. The epididymosomatic index (ESI, g/kg) was also estimated as a ratio of each epididymal weight to body weight. Testicular longitudinal length (TLL) and mid-circumference (TMC) were estimated with a measuring tape. Sperm heads in homogenized testicular and cauda epididymal tissues were counted with a manual cytometer (haemocytometer) as earlier described by Amann and Lambiase [32] and Igbokwe et al. [33] with dilution of homogenates to facilitate counts and dilution factor used in the final calculation of counts. Optimal cauda epididymal sperm reserve was adjudged as ≥1.10 × 10^9^ sperm cells [9, 15].

The data obtained were summarized as means ± standard deviations, means were compared by two-way analysis of variance with Tukey post hoc test, and coefficients (*r*) of correlation were determined using computer software (GraphPad Instat, 1993 version, http://www.graphpad.com/scientific-software/instat/). Correlation coefficients were significant (*p* < 0.05) at >0.30 [34]. 

## 3. Results

Sixty-six (52.80%) out of 125 bucks had normal cauda epididymal sperm cell count of >1.10 × 10^9^, and the data on their ages, BW, testicular (TSC) and cauda epididymal (ESC) sperm counts, and scrotal, testicular, and epididymal size variables are summarized in Table 1. The correlation coefficients in the relationships among the variables are presented in Table 2. The BW of the bucks increased (*p* < 0.05) with age and the sperm counts from the testes and cauda epididymides were higher (*p* < 0.05) at 30 months than 18 and 24 months of age. BW correlated (*r* = 0.49–0.70) with testicular and epididymal size variables (SC, SL, TW, TLL, and TMC) but did not correlate (*r* = 0.15–0.27) significantly (*p* > 0.05) with GSI and sperm counts (TSC, ESC). GSI correlated with TW (*r* = 0.80) and EW (*r* = 0.77) as well as SC, SL, TLL, and TMC (*r* = 0.58–0.80) but failed to correlate significantly (*p* > 0.05) with TSC (*r* = 0.22) and ESC (*r* = 0.25). ESC correlated significantly (*p* < 0.05) with scrotal size variables (SC, SL), some testicular size variables (TW, TLL, and TMC), and EW, whereas TSC did not have significant (*p* > 0.05) correlation with these variables. However, ESC correlated (*r* = 0.55; *p* < 0.05) with TSC as EW also correlated (*r* = 0.92; *p* < 0.05) with TW. The size variables of the scrotum and testis correlated (*r* = 0.70–0.77; *p* < 0.05) with one another.

## 4. Discussion

Majority of the bucks surveyed had optimal ESC and those with lower ESC than the cut-off sperm count were excluded from this report, having been evaluated for hypospermatogenesis and aspermatogenesis as earlier reported [30]. The bucks with optimal and suboptimal ESC had similar age range; the later had lower testicular weights (3.50–54.10 g) and gonadosomatic index values (0.40–2.80 g/kg) than the former [30, 35]. The incidence of testicular hypoplasia and atrophy had been reported recently among our Sahel goat populations [27–29], suggesting the need to determine the appropriate testicular sizes with optimal sperm output. This report provides such data on Sahel bucks with optimal ESC in the semiarid Sahel region and agrees with some limited data earlier reported of some testicular size parameters of mature RS and BW bucks [21]. RS bucks, aged 24–30 months, had mean testicular weight of 83.70 g at mean body weight of 17.80 kg [8]; and the GSI was 4.70 g/kg [35]. At 12–36 months, RS bucks weighed 22.50–30.00 kg with testicular weights of 55.00–103.00 g [21] and GSI of 2.40–3.40 g/kg [35], whereas BW bucks weighed 18.60–28.90 kg with testicular weights of 50.00–100.50 g [21] and GSI of 2.60–3.50 g/kg [35]. In the present study, the testicular weights of Sahel bucks were 31.40–86.20 g where body weights were 12.00–25.00 kg giving the GSI as 2.00–4.50 g/kg.

The age for sexual maturity for Sahel bucks is 3–12 months [11]. The earliest age reported for adequate sperm production was 5 months in WAD bucks [9] and 5.70 months in British bucks [36]. Tropical male goats were reported to reach puberty and sexual maturity at 3.20 and 4.40 months of age, respectively [37], but male Nubian goats reached puberty at 8 months of age [38]. The Sahel bucks, in this study, were expected to have adequate sperm output at >18 months, if the testicular sizes were appropriate and testicular hypoplasia was precluded. As the bucks got older up to 30 months of age, the sperm output was increased because the bucks had increasing BW and testicular size. The testes of Sahel goats grow until mature body weight is attained at ≥30 months of age [39]. Previous reports indicated that testicular size positively correlated with sperm production in bulls, rams, boars, stallions [40, 41], and WAD and cashmere goats [15, 42]. Therefore, the TW and related size parameters would be imperative in selecting sire that is reproductively sound as proposed by Ott and Memon [43]. Scrotal size parameters (SC, SL) correlated with BW, TW, and sperm output in this study, similar to earlier reports [23, 25, 26, 43–48]. The testicular size parameters (TW, GSI, TLL, and TMC) increased with age, indicating that testicular growth was sustained within the period because of expansion of the seminiferous tubular epithelium and associated increase in sperm output, indicating that more mature bucks will have better sperm ejaculation to enhance siring capacity. While BW of the bucks increased with age, BW had low insignificant correlation with TSC and ESC, suggesting that BW variation may not be an absolutely efficient predictor of sperm output in the bucks, in spite of the finding that BW had strong correlation with testicular size (TW, TLL, and TMC) and moderate correlation with scrotal size (SC, SL). The strong correlation between TSC and ESC justifies the dependence of semen sperm count on testicular sperm production and cauda epididymal sperm reserves. However, there was lack of remarkable dependence of testicular sperm count on testicular and related size variables among the goats. Biometric evaluations may establish assumptions of testicular sperm production, but assessment of quality of sperm ejaculates will still be necessary to have enhanced assurance of reproductive soundness of siring Sahel bucks.

In conclusion, the data in this report highlighted a relationship between morphometric parameters associated with size of testis and the sperm reserve that could influence semen quality in terms of sperm output. The impact is anchored on the provision of reference data for the locality on the testicular size measurements which may be useful in prediction of adequate sperm production and normospermia during preliminary selection of sires for breeding purposes.

## Figures and Tables

**Table 1:** Age-related variations in body weights, testicular and epididymal size estimates, and sperm counts in Nigerian Sahel goats.

Parameters	Age (months)			All goats (*n* = 66)	Range (Min.–Max.)
	18 (*n* = 14)	24 (*n* = 25)	30 (*n* = 27)		
Body weight (kg)	14.00 ± 1.13^a^	16.67 ± 2.82^b^	18.74 ± 2.29^c^	17.04 ± 2.99	12.00–25.00
Sperm cell count (×10^9^)					
Cauda epididymal	1.68 ± 0.31^a^	1.68 ± 0.38^a^	2.34 ± 0.80^b^	1.94 ± 0.67	1.12–4.66
Testicular	0.14 ± 0.10^a^	0.15 ± 0.01^a^	0.27 ± 0.20^b^	0.23 ± 0.30	0.03–1.88
Scrotal size					
Length (cm)	8.39 ± 0.68^a^	9.66 ± 2.32^b^	9.89 ± 0.61^b^	9.29 ± 0.91	7.50–11.00
Circumference (cm)	17.82 ± 0.67^a^	18.87 ± 1.22^b^	19.91 ± 1.01^c^	19.07 ± 1.29	17.00–21.80
Testicular size					
Gonadosomatic index (g/kg)	2.85 ± 0.38^a^	3.43 ± 0.67^b^	3.98 ± 0.41^c^	3.51 ± 0.69	2.00–4.50
Weight (g)	39.58 ± 5.73^a^	56.30 ± 11.26^b^	74.34 ± 7.65^c^	59.90 ± 16.10	31.40–86.20
Longitudinal length (cm)	8.94 ± 0.68^a^	10.02 ± 1.07^b^	11.13 ± 0.79^c^	10.23 ± 1.20	7.80–12.90
Mid-circumference (cm)	10.23 ± 0.65^a^	11.36 ± 0.87^b^	12.71 ± 0.62^c^	11.57 ± 1.30	9.10–13.70
Epididymal size					
Weight (g)	6.46 ± 1.17^a^	8.15 ± 1.53^b^	10.11 ± 0.69^c^	8.57 ± 1.82	4.60–11.70
Epididymal-testicular weight ratio (g/g)	0.16 ± 0.02^a^	0.15 ± 0.02^a^	0.13 ± 0.01^b^	0.15 ± 0.02	0.11–0.19
Epididymosomatic index (g/kg)	0.46 ± 0.08^a^	0.49 ± 0.10^ab^	0.54 ± 0.05^b^	0.51 ± 0.08	0.28–0.64

^a,b,c^Means ± standard deviation with different superscripts are significantly different (*p* < 0.05).

**Table 2:** Correlation coefficients^*∗*^ (*r*) in matrix of relationships among testicular and related variables and associated sperm counts.

Variables	GSI	SC	SL	TW	TLL	TMC	EW	ESC	TSC	BW
Gonadosomatic index (GSI)	1									
Scrotal circumference (SC)	0.58	1								
Scrotal length (SL)	0.59	0.84	1							
Testicular weight (TW)	0.80	0.77	0.72	1						
Testicular longitudinal length (TLL)	0.60	0.74	0.70	0.89	1					
Testicular mid-circumference (TMC)	0.76	0.77	0.76	0.96	0.89	1				
Epididymal weight (EW)	0.77	0.68	0.64	0.92	0.83	0.89	1			
Cauda epididymal sperm count (ESC)	0.25	0.34	0.30	0.36	0.35	0.37	0.39	1		
Testicular sperm count (TSC)	0.22	0.15	0.20	0.16	0.12	0.17	0.21	0.55	1	
Body weight (BW)	0.15	0.60	0.49	0.70	0.76	0.69	0.60	0.27	0.04	1

^*∗*^ *r* ≥ 0.3 is significant (*p* < 0.05).
